# Supplementary material for: The CRISPR-Cas13a Gemini System for noncontiguous target RNA activation
Source: Nat Commun. 2024 Apr 4;15:2901. doi: 10.1038/s41467-024-47281-w (PMC10994916; doi:10.1038/s41467-024-47281-w)
Supplement: Supplementary file 1 — Supplementary Information [file 41467_2024_47281_MOESM1_ESM.pdf]

Supplementary information for "The CRISPR-Cas13a Gemini System for noncontiguous target RNA activation"

Hongrui Zhao[1,2,†], Yan Sheng[2,3,†,*], Tenghua Zhang[2], Shujun Zhou[2], Yuqing Zhu[2], Feiyang Qian[2], Meiru Liu[1], Weixue Xu[1], Dengsong Zhang[1,**], Jiaming Hu[1,2,***]

[1] International Joint Laboratory of Catalytic Chemistry, State Key Laboratory of Advanced Special Steel, Innovation Institute of Carbon Neutrality, College of Sciences, Shanghai University, Shanghai 200444, China

[2] MOE Key Laboratory of Laser Life Science & Institute of Laser Life Science, Guangdong Provincial Key Laboratory of Laser Life Science, College of Biophotonics, South China Normal University, Guangzhou 510631, China.

[3] Institute of Translational Medicine, Shanghai University, Shanghai 200444, China.

To whom correspondence should be addressed: ysheng@shu.edu.cn (Y. Sheng), dszhang@shu.edu.cn (D. Zhang), jmhu@shu.edu.cn (J. Hu)

[†] These authors contributed equally.

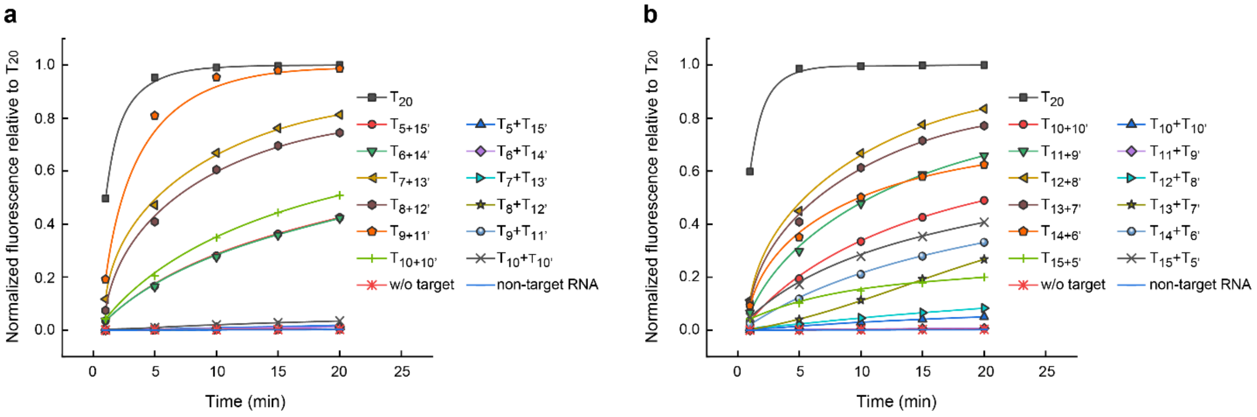

Supplementary Fig. 1 Quantification of fluorescence signal generated by Cas13a HEPN-nuclease activation in the presence of 11 groups of target RNAs. a, Representative time course of fluorescence measurements was generated by Cas13a HEPN-nuclease activation by the addition of G_{5-15} to G_{10-10}. **b**, Representative time course of fluorescence measurements was generated by Cas13a HEPN-nuclease activation by the addition of G_{10-10} to G_{15-5}.

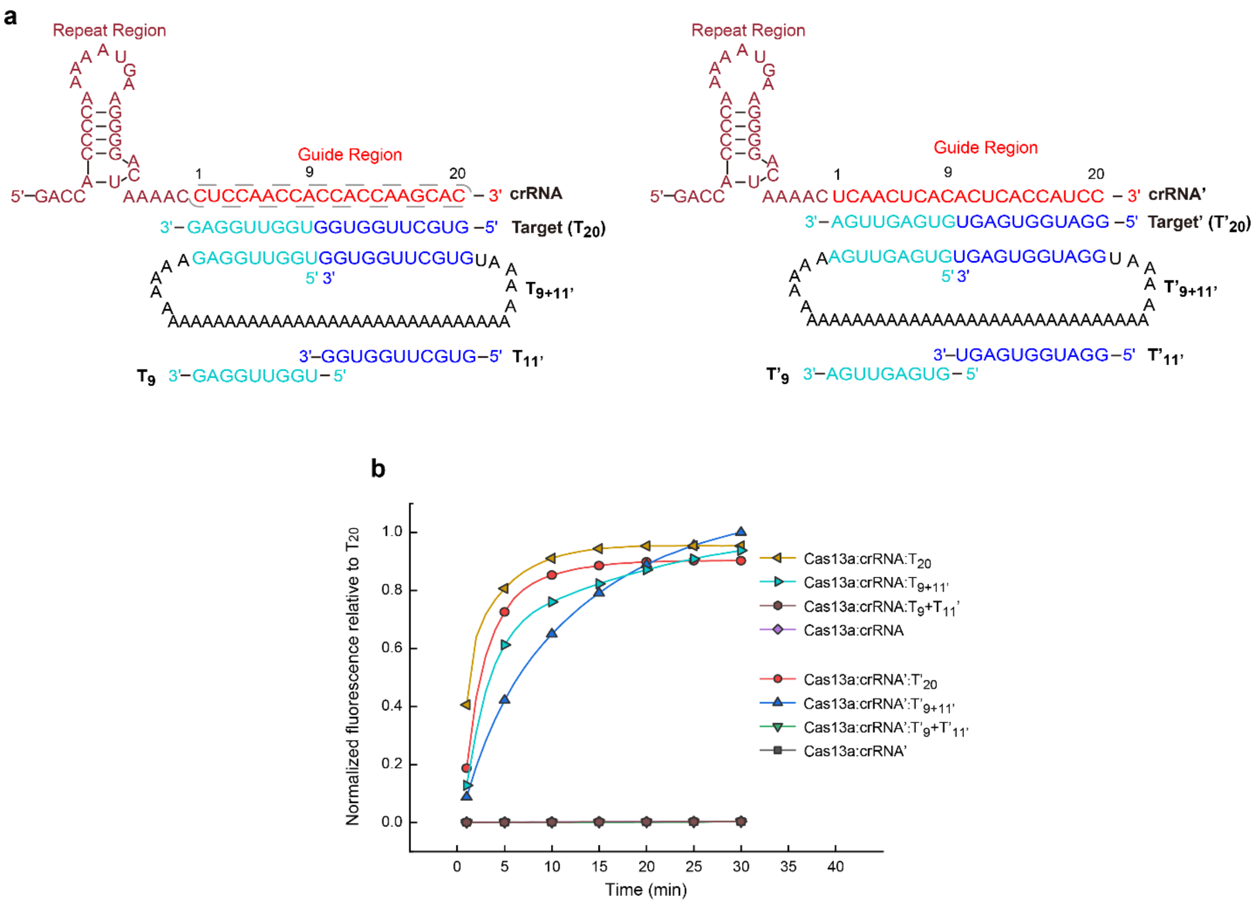

Supplementary Fig. 2 Fluorescence analysis of the activation of Cas13a collateral cleavage activity by $G_{9\text{-}11}$. a, Sequence information of crRNA, $T_{9+11'}$, T_9+$T_{11'}$, crRNA', $T'_{9+11'}$ and T'_9+$T'_{11'}$. **b,** Fluorescence measurement of Cas13a activated by $G_{9\text{-}11}$.

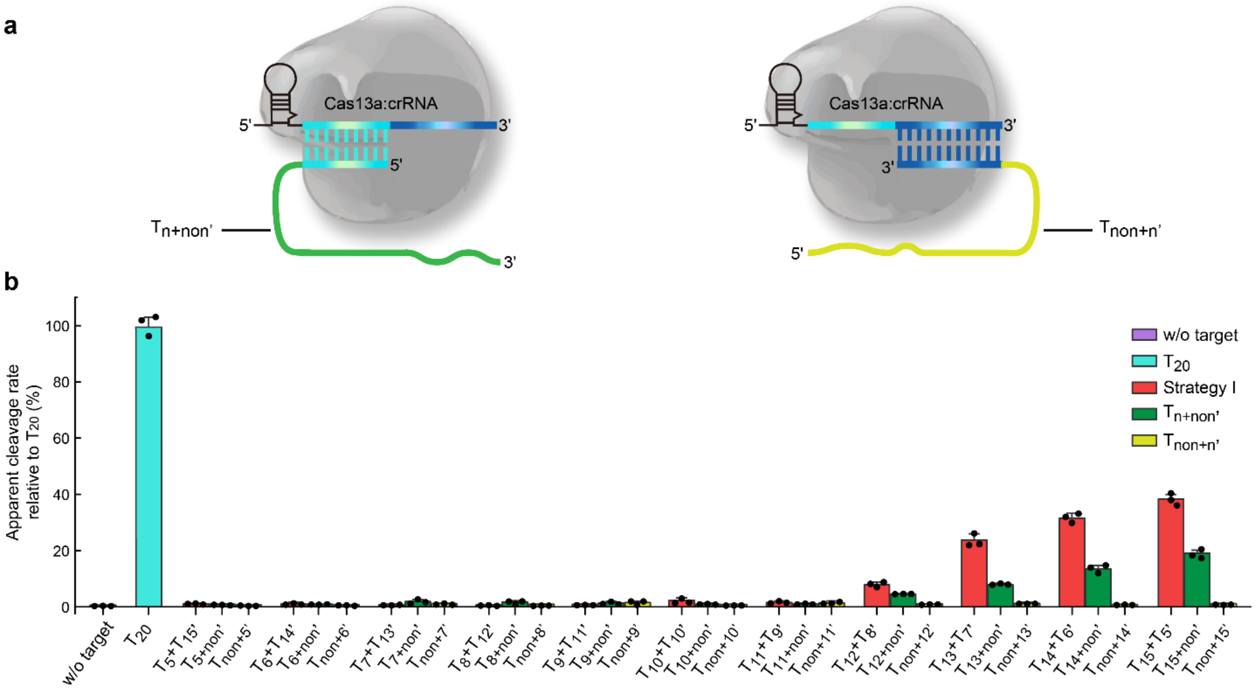

Supplementary Fig. 3 RNA activator controls that possess a single crRNA targeting region. **a**, Schematic of RNA activator controls ($T_{n+non'}$, $T_{non+n'}$). **b**, Apparent cleavage rate of RNA activator controls ($T_{n+non'}$, $T_{non+n'}$) for Cas13a trans-cleavage relative to T_{20}. Data are presented as mean values ± standard deviation from three independent experiments.

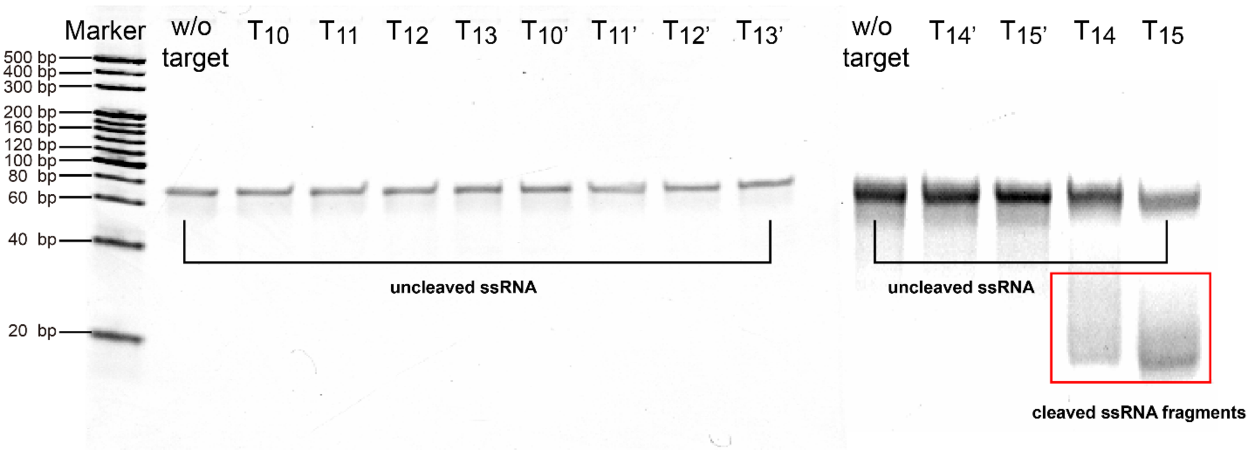

Supplementary Fig. 4 Electrophoretic gel analysis of Cas13a activation by target RNAs of 10 to 15-nt in length. 10 to 13-nt-target RNAs failed to activate Cas13a which cannot cleave single-stranded RNA (ssRNA) to produce fragmented products (left). Besides, target RNA shortened by 5-nt or more from its 3' end ($T_{15'}$ or $T_{14'}$) also failed to activate Cas13a (right). The experiment was repeated twice independently with similar results.

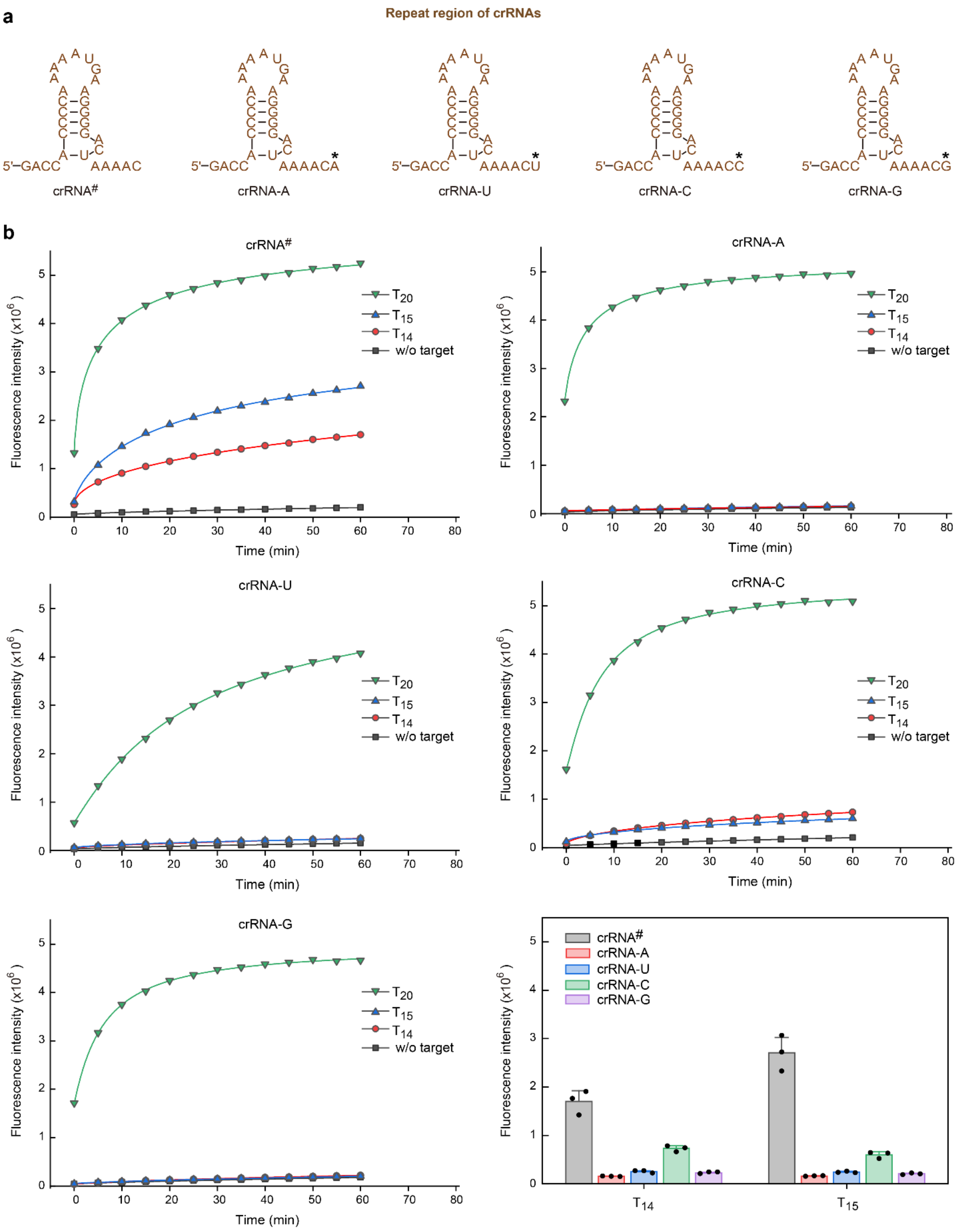

Supplementary Fig. 5 Difference in crRNA sequence. a, Sequences of the designed crRNAs

(crRNA$^{\#}$, crRNA-A, crRNA-U, crRNA-C, crRNA-G) with the same guide region but different in repeat region. **b**, Fluorescence analysis of Cas13a activation with designed crRNAs by T_{14} and T_{15}, respectively. Data are presented as mean values ± standard deviation from three independent experiments.

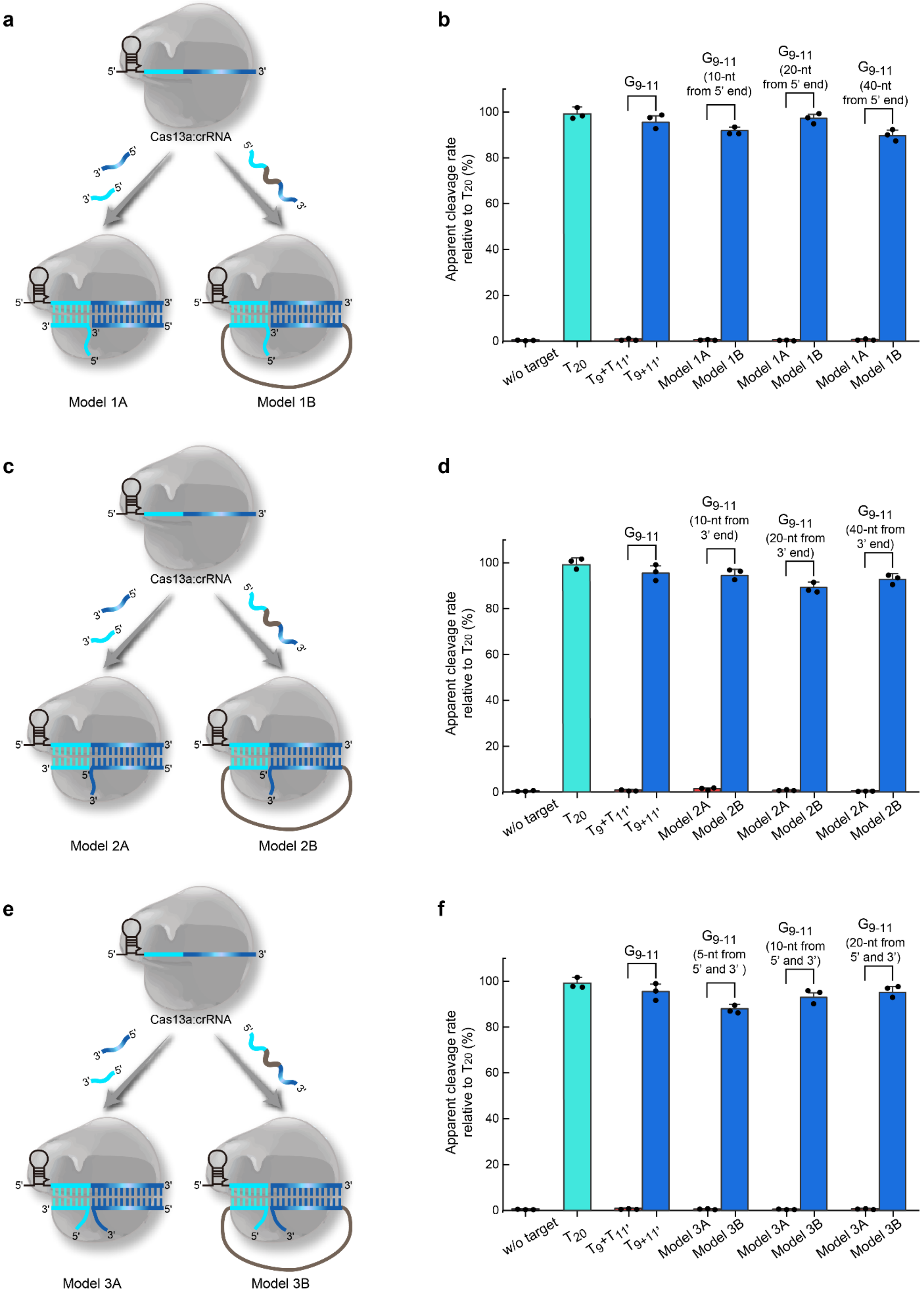

a
Cas13a:crRNA
Model 1A
Model 1B
b
Apparent cleavage rate relative to T20 (%)
G9-11
G9-11 (10-nt from 5' end)
G9-11 (20-nt from 5' end)
G9-11 (40-nt from 5' end)
w/o target
T20
T9+T11'
T9+11'
Model 1A
Model 1B
c
Cas13a:crRNA
Model 2A
Model 2B
d
Apparent cleavage rate relative to T20 (%)
G9-11
G9-11 (10-nt from 3' end)
G9-11 (20-nt from 3' end)
G9-11 (40-nt from 3' end)
w/o target
T20
T9+T11'
T9+11'
Model 2A
Model 2B
e
Cas13a:crRNA
Model 3A
Model 3B
f
Apparent cleavage rate relative to T20 (%)
G9-11
G9-11 (5-nt from 5' and 3')
G9-11 (10-nt from 5' and 3')
G9-11 (20-nt from 5' and 3')
w/o target
T20
T9+T11'
T9+11'
Model 3A
Model 3B

Supplementary Fig. 6 Noncontiguous target RNA activation paradigm of Cas13a. a, Schematic of model 1 for activating Cas13a. **b**, Apparent cleavage rate of model 1 for Cas13a trans-cleavage relative to T_{20}. **c**, Schematic of model 2 for activating Cas13a. **d**, Apparent cleavage rate of model 2 for Cas13a trans-cleavage relative to T_{20}. **e**, Schematic of model 3 for activating Cas13a. **f**, Apparent cleavage rate of model 3 for Cas13a trans-cleavage relative to T_{20}. Data are presented as mean values ± standard deviation from three independent experiments.

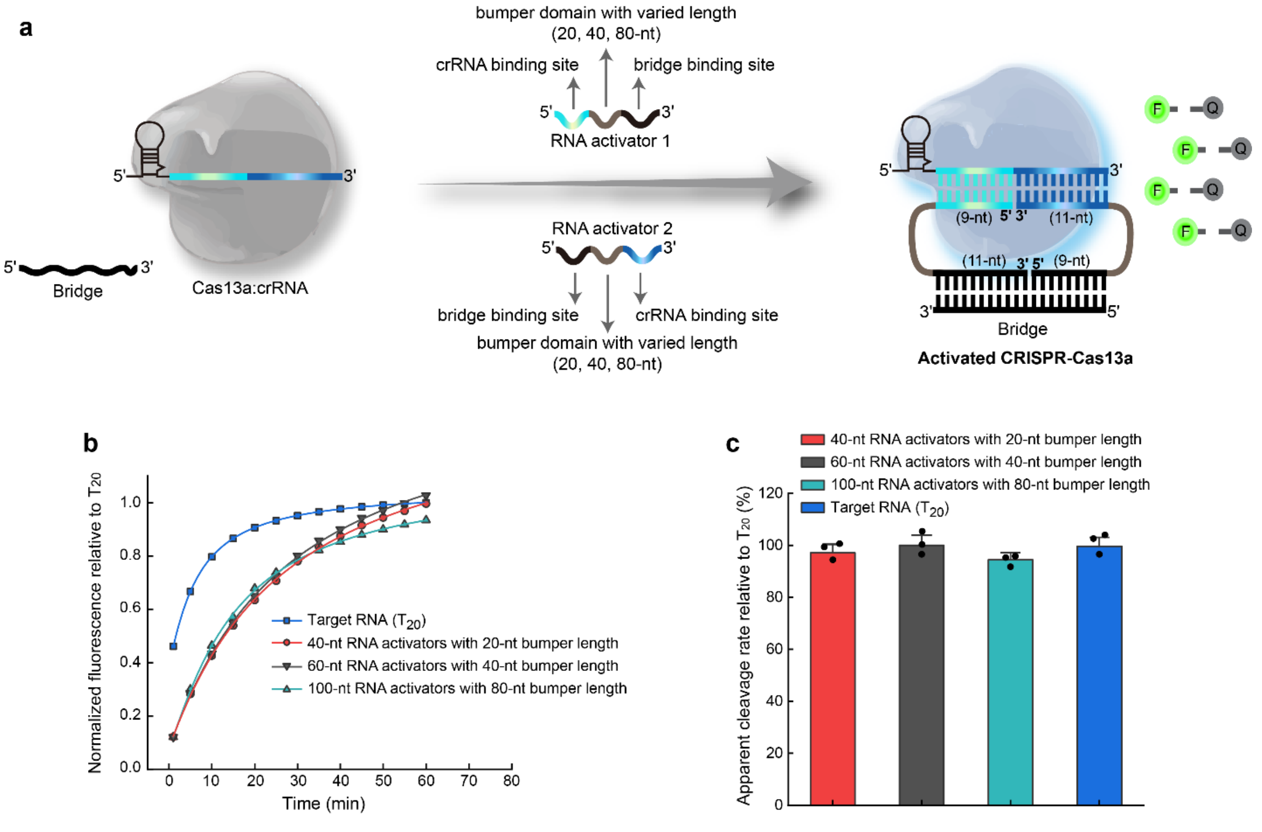

Supplementary Fig. 7 Two RNA activators co-activate Cas13a with the assistance of an auxiliary sequence. a, Schematic of two RNA activators with varied bumper length (20, 40, 80-nt) co-activate Cas13a with the assistance of an auxiliary sequence. **b**, Fluorescence analysis of HEPN-nuclease activity of Cas13a by two 40-, 60- or 100-nt RNA activators. **c**, Apparent cleavage rate of two 40-, 60- or 100-nt RNA activators-mediated Cas13a trans-cleavage relative to contiguous target RNA (T_{20}). Data are presented as mean values ± standard deviation from three independent experiments.

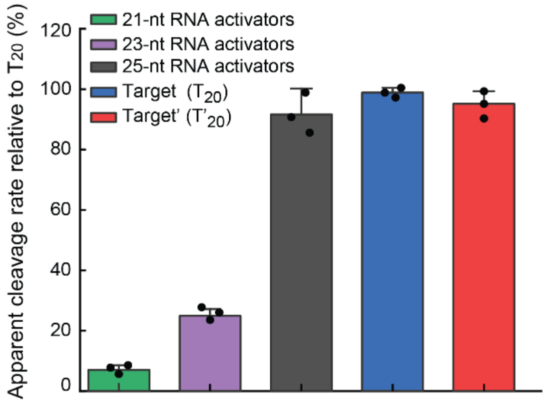

Supplementary Fig. 8 Apparent cleavage rate of two 21-, 23- or 25-nt RNA activators-mediated Cas13a trans-cleavage reporter relative to contiguous Target (T_{20}). Data are presented as mean values ± standard deviation from three independent experiments.

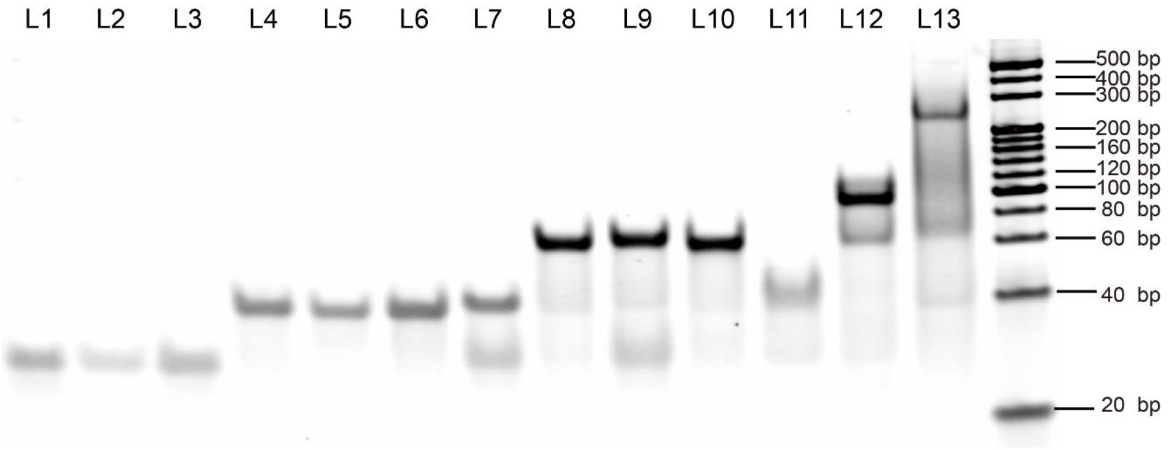

L1. 25-nt RNA activator 1
L2. 25-nt RNA activator 2
L3. 25-nt RNA activator 1+2
L4. crRNA
L5. crRNA'
L6. crRNA+crRNA'
L7. crRNA'+25-nt RNA activator 1
L8. crRNA'+25-nt RNA activator 2
L9. crRNA'+25-nt RNA activator 1+2
L10. crRNA+25-nt RNA activator 1
L11. crRNA+25-nt RNA activator 2
L12. crRNA+25-nt RNA activator 1+2
L13. crRNA+crRNA'+25-nt RNA activator 1+2

Supplementary Fig. 9 Electrophoretic analysis of the formation processing of crRNA:RNA activator 1:RNA activator 2:crRNA' quaternary complex. The experiment was repeated twice independently with similar results.

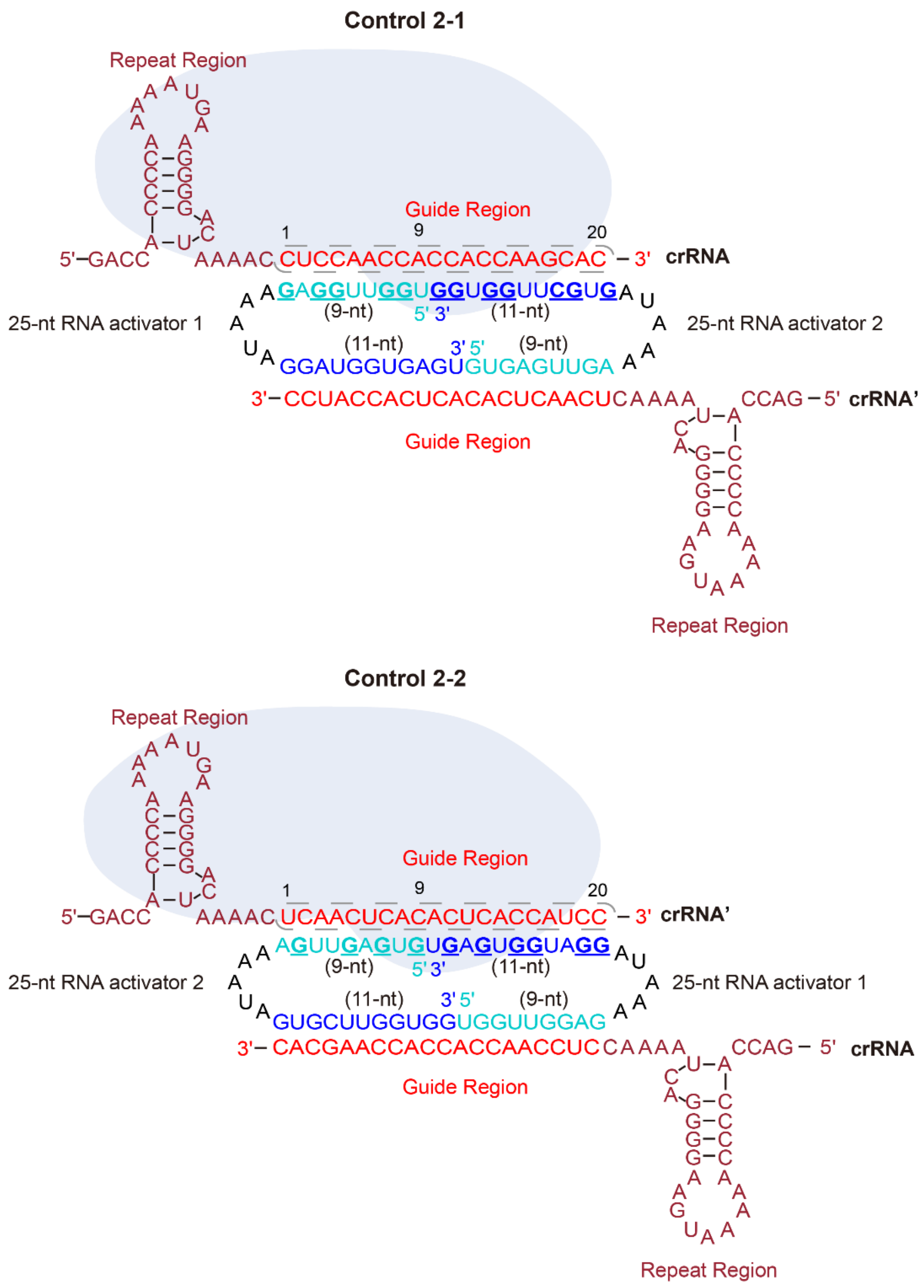

Supplementary Fig. 10 Schematic of the quinary complexes of Control 2-1 and Control 2-2.

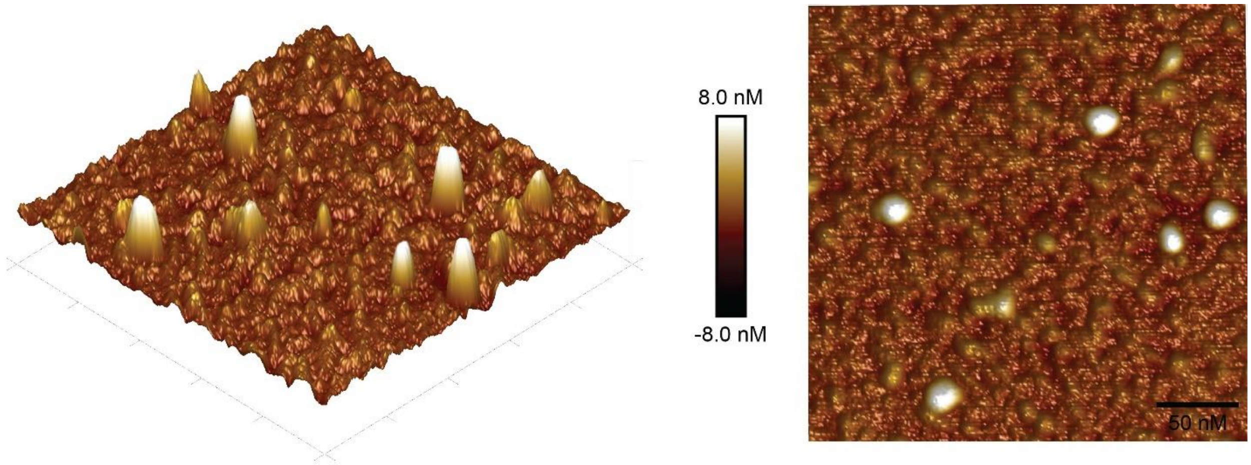

Supplementary Fig. 11 3D and 2D AFM images of Control 2-1 or Control 2-2. The experiment was repeated twice independently with similar results.

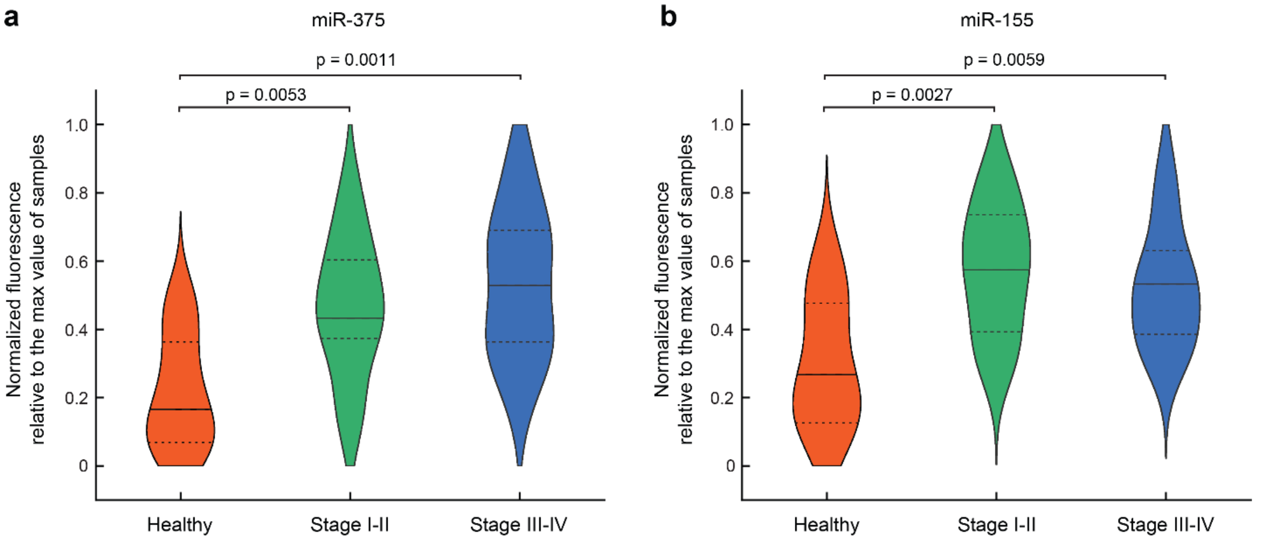

Supplementary Fig. 12 Measurements of single miRNA markers in serum samples. a, Statistical quantification of miR-375 expression in the serum of healthy subjects, stage I-II and III-IV BC patients. **b,** Statistical quantification of miR-155 expression in the serum of healthy subjects, stage I-II and III-IV BC patients. Violin plots are centered around the median (black line) with quartiles (lower and upper dashed lines). Minima and maxima are shown as the bottom and top of the violin plots, respectively. Two-tailed t-test was used for statistical analysis.

a

b

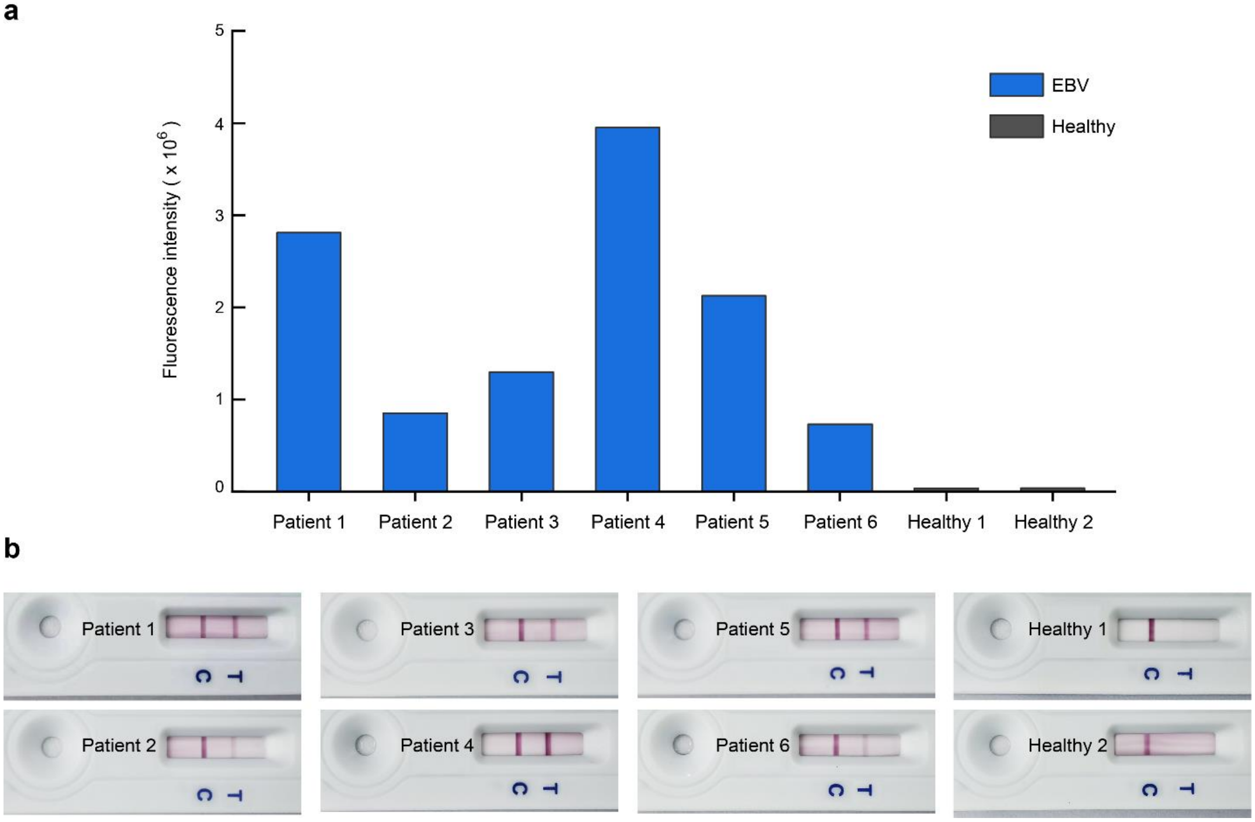

Supplementary Fig. 13 Comparison of CRISPR-Cas13a Gemini System in fluorescence assay with commercial LFA in clinical sample detection. **a**, Fluorescence intensities of CRISPR-Cas13a Gemini System. **b**, The photos of LFA.

Supplementary Table 1 Oligonucleotides used in this work.

Name	Abbreviate	Sequence 5'-3'
crRNA		GACCACCCCAAAAAUGAAGGGGACUAAAACCUCCAACCACCACCAAGCAC
crRNA'		GACCACCCCAAAAAUGAAGGGGACUAAAACUCAACUCACACUCACCAUCC
Target	T_{20}	GUGCUUGGUGGUGGUUGGAG
Target'	T'_{20}	GGAUGGUGAGUGUGAGUUGA
Target 5	T5	UGGAG
Target 15'	T15'	GUGCUUGGUGGUGGU
Target 5+15'	$T_{5+15'}$	UGGAGAAAAAAAAAAAAAAAAAAAAAAAAAAAAAAAAAAAAAAAAUGUGCUUGGUGGUGGU
Target 6	T6	UUGGAG
Target 14'	T14'	GUGCUUGGUGGUGG
Target 6+14'	$T_{6+14'}$	UUGGAGAAAAAAAAAAAAAAAAAAAAAAAAAAAAAAAAAAAAAAAAUGUGCUUGGUGGUGG
Target 7	T7	GUUGGAG
Target 13'	T13'	GUGCUUGGUGGUG
Target 7+13'	$T_{7+13'}$	GUUGGAGAAAAAAAAAAAAAAAAAAAAAAAAAAAAAAAAAAAAAAAAUGUGCUUGGUGGUG
Target 8	T8	GGUUGGAG
Target 12'	T12'	GUGCUUGGUGGU
Target 8+12'	$T_{8+12'}$	GGUUGGAGAAAAAAAAAAAAAAAAAAAAAAAAAAAAAAAAAAAAAAAAUGUGCUUGGUGGU
Target 9	T9	UGGUUGGAG
Target 11'	T11'	GUGCUUGGUGG
Target 9+11'	$T_{9+11'}$	UGGUUGGAGAAAAAAAAAAAAAAAAAAAAAAAAAAAAAAAAAAAAAAAAUGUGCUUGGUGG
Target 10	T10	GUGGUUGGAG
Target 10'	T10'	GUGCUUGGUG
Target 10+10'	$T_{10+10'}$	GUGGUUGGAGAAAAAAAAAAAAAAAAAAAAAAAAAAAAAAAAAAAAAAAAUGUGCUUGGUG
Target 11	T11	GGUGGUUGGAG
Target 9'	T9'	GUGCUUGGU
Target 11+9'	$T_{11+9'}$	GGUGGUUGGAGAAAAAAAAAAAAAAAAAAAAAAAAAAAAAAAAAAAAAAAAUGUGCUUGGU
Target 12	T12	UGGUGGUUGGAG
Target 8'	T8'	GUGCUUGG
Target 12+8'	$T_{12+8'}$	UGGUGGUUGGAGAAAAAAAAAAAAAAAAAAAAAAAAAAAAAAAAAAAAAAAAUGUGCUUGG
Target 13	T13	GUGGUGGUUGGAG
Target 7'	T7'	GUGCUUG
Target 13+7'	$T_{13+7'}$	GUGGUGGUUGGAGAAAAAAAAAAAAAAAAAAAAAAAAAAAAAAAAAAAAAAAAUGUGCUUG
Target 14	T14	GGUGGUGGUUGGAG
Target 6'	T6'	GUGCUU
Target 14+6'	$T_{14+6'}$	GGUGGUGGUUGGAGAAAAAAAAAAAAAAAAAAAAAAAAAAAAAAAAAAAAAAAAUGUGCUU
Target 15	T15	UGGUGGUGGUUGGAG
Target 5'	T5'	GUG CU
Target 15+5'	$T_{15+5'}$	UGGUGGUGGUUGGAGAAAAAAAAAAAAAAAAAAAAAAAAAAAAAAAAAAAAAAAAUGUGCU
Target' 9	T'9	GUGAGUUGA
Target' 11'	T'11'	GGAUGGUGAGU
Target' 9+11'	$T'_{9+11'}$	GUGAGUUGAAAAAAAAAAAAAAAAAAAAAAAAAAAAAAAAAAAAAAAAAUGGAUGGUGAGU
21-nt RNA activator 1		UGGUUGGAGAGGAUGGUGAGU
21-nt RNA activator 2		GUGAGUUGAAGUGCUUGGUGG
23-nt RNA activator 1		UGGUUGGAGAAAGGAUGGUGAGU
23-nt RNA activator 2		GUGAGUUGAAAAGUGCUUGGUGG
25-nt RNA activator 1		UGGUUGGAGAAAUAGGAUGGUGAGU
25-nt RNA activator 2		GUGAGUUGA AAAUAGUGCUUGGUGG
FQ5U		/FAM/UUUUU/BHQ1/
non-target RNA		GGAUGGUGAGUGUGAGUUGA
RNA substrate		GAUUUAGACUACCCCAAAAACGAAGGGGACUAAAACGGUUUUUUUUUUUUUUUUUUUUGGGGGG

Supplementary Table 2 Oligonucleotides used in Supplementary Fig. 3.

Name	Sequence 5'-3'
T_{20}	GUGCUUGGUGGUGGUUGGAG
T5	UGGAG
T15'	GUGCUUGGUGGUGGU
$T_{5+non'}$	UGGAGAAAAAAAAAAAAAAAAAAAAAAAAAAAAAAAAAAAAAAAUAAAAAAAAAAAAAAA
$T_{non+5'}$	AAAAAAAAAAAAAAAAAAAAAAAAAAAAAAAAAAAAAAAAAAAAAAAAAAAAAAUGUGCU
T6	UUGGAG
T14'	GUGCUUGGUGGUGG
$T_{6+non'}$	UUGGAGAAAAAAAAAAAAAAAAAAAAAAAAAAAAAAAAAAAAAAAUAAAAAAAAAAAAAA
$T_{non+6'}$	AAAAAAAAAAAAAAAAAAAAAAAAAAAAAAAAAAAAAAAAAAAAAAAAAAAAAUGUGCUU
T7	GUUGGAG
T13'	GUGCUUGGUGGUG
$T_{7+non'}$	GUUGGAGAAAAAAAAAAAAAAAAAAAAAAAAAAAAAAAAAAAAAAAUAAAAAAAAAAAAA
$T_{non+7'}$	AAAAAAAAAAAAAAAAAAAAAAAAAAAAAAAAAAAAAAAAAAAAAAAAAAAAUGUGCUUG
T8	GGUUGGAG
T12'	GUGCUUGGUGGU
$T_{8+non'}$	GGUUGGAGAAAAAAAAAAAAAAAAAAAAAAAAAAAAAAAAAAAAAAAUAAAAAAAAAAAA
$T_{non+8'}$	AAAAAAAAAAAAAAAAAAAAAAAAAAAAAAAAAAAAAAAAAAAAAAAAAAAUGUGCUUGG
T9	UGGUUGGAG
T11'	GUGCUUGGUGG
$T_{9+non'}$	UGGUUGGAGAAAAAAAAAAAAAAAAAAAAAAAAAAAAAAAAAAAAAAAUAAAAAAAAAAA
$T_{non+9'}$	AAAAAAAAAAAAAAAAAAAAAAAAAAAAAAAAAAAAAAAAAAAAAAAAAAUGUGCUUGGU
T10	GUGGUUGGAG
T10'	GUGCUUGGUG
$T_{10+non'}$	GUGGUUGGAGAAAAAAAAAAAAAAAAAAAAAAAAAAAAAAAAAAAAAAAUAAAAAAAAAA
$T_{non+10'}$	AAAAAAAAAAAAAAAAAAAAAAAAAAAAAAAAAAAAAAAAAAAAAAAAAUGUGCUUGGUG
T11	GGUGGUUGGAG
T9'	GUGCUUGGU
$T_{11+non'}$	GGUGGUUGGAGAAAAAAAAAAAAAAAAAAAAAAAAAAAAAAAAAAAAAAAUAAAAAAAAA
$T_{non+11'}$	AAAAAAAAAAAAAAAAAAAAAAAAAAAAAAAAAAAAAAAAAAAAAAAAUGUGCUUGGUGG
T12	UGGUGGUUGGAG
T8'	GUGCUUGG
$T_{12+non'}$	UGGUGGUUGGAGAAAAAAAAAAAAAAAAAAAAAAAAAAAAAAAAAAAAAAAUAAAAAAAA
$T_{non+12'}$	AAAAAAAAAAAAAAAAAAAAAAAAAAAAAAAAAAAAAAAAAAAAAAAUGUGCUUGGUGGU
T13	GUGGUGGUUGGAG
T7'	GUGCUUG
$T_{13+non'}$	GUGGUGGUUGGAGAAAAAAAAAAAAAAAAAAAAAAAAAAAAAAAAAAAAAAAUAAAAAAA
$T_{non+13'}$	AAAAAAAAAAAAAAAAAAAAAAAAAAAAAAAAAAAAAAAAAAAAAAUGUGCUUGGUGGUG
T14	GGUGGUGGUUGGAG
T6'	GUGCUU
$T_{14+non'}$	GGUGGUGGUUGGAGAAAAAAAAAAAAAAAAAAAAAAAAAAAAAAAAAAAAAAAUAAAAAA
$T_{non+14'}$	AAAAAAAAAAAAAAAAAAAAAAAAAAAAAAAAAAAAAAAAAAAAAUGUGCUUGGUGGUGG
T15	UGGUGGUGGUUGGAG
T5'	GUG CU
$T_{15+non'}$	UGGUGGUGGUUGGAGAAAAAAAAAAAAAAAAAAAAAAAAAAAAAAAAAAAAAAAUAAAAA
$T_{non+15'}$	AAAAAAAAAAAAAAAAAAAAAAAAAAAAAAAAAAAAAAAAAAAAUGUGCUUGGUGGUGGU

Supplementary Table 3 Oligonucleotides used in Fig. 4 and 5.

Name	Sequence 5'-3'
Sequences used in CRISPR-Cas13a Gemini System for breast cancer diagnosis:	
crRNA	GACCACCCCAAAAAUGAAGGGGACUAAAACACGAACAAAAACCCCUAUCA
crRNA'	GACCACCCCAAAAAUGAAGGGGACUAAAACUAGCAUUAAUCACGCGAGCC
miR-375	UUUGUUCGUUCGGCUCGCGUGA
miR-155	UUAAUGCUAAUCGUGAUAGGGGUU
Sequences used in CRISPR-Cas13a Gemini System for EBV diagnosis:	
crRNA	GACCACCCCAAAAAUGAAGGGGACUAAAACGUAGGUCCUAUAGCGGACAA
crRNA'	GACCACCCCAAAAAUGAAGGGGACUAAAACGGCUGUCCUCAUGCGGACCA
EBER-1	AGGACCUACGCUGCCCUAGAGGUUUUGCUAGGGAGGAGACGUGUGUGGCUGUAGCC<br>ACCCGUCCCGGGUACAAGUCCCGGGUGGUGAGGACGGUGUCUGUGGUUGUCUUCCC<br>AGACUCUGCUUUCUGCCGUCUUCGGUCAAGUACCAGCUGGUGGUCCGCAUGUUUU
EBER-2	AGGACAGCCGUUGCCCUAGUGGUUUCGGACACACCGCCAACGCUCAGUGCGGUGCUA<br>CCGACCCGAGGUCAAGUCCCGGGGGAGGAGAAGAGAGGCUUCCCGCCUAGAGCAUUUG<br>CAAGUCAGGAUUCUCUAAUCCCUCUGGGAGAAGGGUAUUCGGCUUGUCCGCUAUUUUU
Sequences used in CRISPR-Cas13a Gemini System for dual transgene knockdown:	
crRNA	GACCACCCCAAAAAUGAAGGGGACUAAAACGCUCACCAUGAGAGUGAUCC
crRNA'	GACCACCCCAAAAAUGAAGGGGACUAAAACGUGAACAGCGCCGGUGGAGU
mCherry	AUGGUGAGCAAGGGCGAGGAGGAUAACAUGGCCAUCAUCAAGGAGUUCAUGCGCUUCA<br>AGGUGCACAUGGAGGGCUCCGUGAACGGCCACGAGUUCGAGAUCGAGGGCGAGGGCG<br>AGGGCCGCCCCUACGAGGGCACCCAGACCGCCAAGCUGAAGGUGACCAAGGGUGGCCC<br>CCUGCCCUUCGCCUGGGACAUCCUGUCCCCUCAGUUCAUGUACGGCUCCAAGGCCUAC<br>GUGAAGCACCCCGCCGACAUCCCCGACUACUUGAAGCUGUCCUUCCCCGAGGGCUUCA<br>AGUGGGAGCGCGUGAUGAACUUCGAGGACGGCGGCGUGGUGACCGUGACCCAGGACU<br>CCUCCCUGCAGGACGGCGAGUUCAUCUACAAGGUGAAGCUGCGCGGCACCAACUUCCC<br>CUCCGACGGCCCCGUAAUGCAGAAGAAGACCAUGGGCUGGGAGGCCUCCUCCGAGCGG<br>AUGUACCCCGAGGACGGCGCCCUGAAGGGCGAGAUCAAGCAGAGGCUGAAGCUGAAGG<br>ACGGCGGCCACUACGACGCUGAGGUCAAGACCACCUACAAGGCCAAGAAGCCCGUGCAG<br>CUGCCCGGCGCCUACAACGUCAACAUCAAGUUGGACAUCACCUCCCACAACGAGGACUA<br>CACCAUCGUGGAACAGUACGAACGCGCCGAGGGCCGCCACUCCACCGGCGGCAUGGAC<br>GAGCUGUACAAG
EGFP	GUGAGCAAGGGCGAGGAGCUGUUCACCGGGGUGGUGCCCAUCCUGGUCGAGCUGGACG<br>GCGACGUAAACGGCCACAAGUUCAGCGUGUCCGGCGAGGGCGAGGGCGAUGCCACCUAC<br>GGCAAGCUGACCCUGAAGUUCAUCUGCACCACCGGCAAGCUGCCCGUGCCCUGGCCCAC<br>CCUCGUGACCACCCUGACCUACGGCGUGCAGUGCUUCAGCCGCUACCCCGACCACAUGAA<br>GCAGCACGACUUCUUCAAGUCCGCCAUGCCCGAAGGCUACGUCCAGGAGCGCACCAUCUU<br>CUUCAAGGACGACGGCAACUACAAGACCCGCGCCGAGGUGAAGUUCGAGGGCGACACCCU<br>GGUGAACCGCAUCGAGCUGAAGGGCAUCGACUUCAAGGAGGACGGCAACAUCCUGGGGCA<br>CAAGCUGGAGUACAACUACAACAGCCACAACGUCUAUAUCAUGGCCGACAAGCAGAAGAAC<br>GGCAUCAAGGUGAACUUCAAGAUCCGCCACAACAUCGAGGACGGCAGCGUGCAGCUCGCC<br>GACCACUACCAGCAGAACACCCCCAUCGGCGACGGCCCCGUGCUGCUGCCCGACAACCAC<br>UACCUGAGCACCCAGUCCGCCCUGAGCAAAGACCCCAACGAGAAGCGCGAUCACAUGGUC<br>CUGCUGGAGUUCGUGACCGCCGCCGGGAUCACUCUCGGCAUGGACGAGCUGUACAAGUAA

The underlined RNA sequences are complementary to the guide regions of crRNA and crRNA'.

Supplementary Table 4 Demographics of healthy subjects and BC patients.

	Healthy subjects	BC patients	
		Stage I-II	Stage III-IV
Number	15	15	15
Female	15	15	15
Median age	46	48	52
Age range	32-65	35-70	37-75

Supplementary Table 5 Table associated with ROC analysis depicted in Fig. 4c.

(Stage I-II vs. healthy)

Target	AUC	Cut-off value	Sensitivity	Specificity	95% CI
miR-375	0.827	≤ 0.368	0.800	0.800	0.679 - 0.974
miR-155	0.853	≤ 0.385	0.800	0.667	0.721 - 0.986
CRISPR-Cas13a Gemini System	0.951	≤ 0.443	0.867	0.933	0.876 - 1.000

(Stage I-IV vs. healthy)

Target	AUC	Cut-off value	Sensitivity	Specificity	95% CI
miR-375	0.851	≤ 0.369	0.767	0.800	0.736 - 0.967
miR-155	0.822	≤ 0.381	0.800	0.667	0.688 - 0.956
CRISPR-Cas13a Gemini System	0.964	≤ 0.463	0.867	1.000	0.919 - 1.000

Supplementary Table 6 Table associated with ROC analysis depicted in Fig. 4f.

Target	AUC	Cut-off value	Sensitivity	Specificity	95% CI
CRISPR-Cas13a Gemini System	0.998	≤ 0.329	1.000	0.962	0.995 - 1.000
