# Supplementary material for: The CRISPR-Cas13a Gemini System for noncontiguous target RNA activation
Source: Nat Commun. 2024 Apr 4;15:2901. doi: 10.1038/s41467-024-47281-w (PMC10994916; doi:10.1038/s41467-024-47281-w)
Supplement: Supplementary file 3 — Reporting summary [file 41467_2024_47281_MOESM3_ESM.pdf]

nature portfolio

Corresponding author(s): Yan Sheng, Dengsong Zhang, Jiaming Hu

Last updated by author(s): Mar 4, 2024

Reporting Summary

Nature Portfolio wishes to improve the reproducibility of the work that we publish. This form provides structure for consistency and transparency in reporting. For further information on Nature Portfolio policies, see our Editorial Policies and the Editorial Policy Checklist.

Statistics

For all statistical analyses, confirm that the following items are present in the figure legend, table legend, main text, or Methods section.

n/a	Confirmed	
☐	☒	The exact sample size (*n*) for each experimental group/condition, given as a discrete number and unit of measurement
☐	☒	A statement on whether measurements were taken from distinct samples or whether the same sample was measured repeatedly
☐	☒	The statistical test(s) used AND whether they are one- or two-sided<br>*Only common tests should be described solely by name; describe more complex techniques in the Methods section.*
☒	☐	A description of all covariates tested
☒	☐	A description of any assumptions or corrections, such as tests of normality and adjustment for multiple comparisons
☐	☒	A full description of the statistical parameters including central tendency (e.g. means) or other basic estimates (e.g. regression coefficient) AND variation (e.g. standard deviation) or associated estimates of uncertainty (e.g. confidence intervals)
☐	☒	For null hypothesis testing, the test statistic (e.g. *F*, *t*, *r*) with confidence intervals, effect sizes, degrees of freedom and *P* value noted<br>*Give P values as exact values whenever suitable.*
☒	☐	For Bayesian analysis, information on the choice of priors and Markov chain Monte Carlo settings
☒	☐	For hierarchical and complex designs, identification of the appropriate level for tests and full reporting of outcomes
☒	☐	Estimates of effect sizes (e.g. Cohen's *d*, Pearson's *r*), indicating how they were calculated

Our web collection on statistics for biologists contains articles on many of the points above.

Software and code

Policy information about availability of computer code

Data collection	The fluorescence data were recorded by Quant Studio 3 (ABI Thermo Fisher Scientific). Gel images were collected by Gel Doc XR+ (Bio-Rad). AFM image was taken on Bruker Dimension Icon AFM. Confocal image were collected using confocal laser scanning microscopy (AX, Nikon).
Data analysis	Statistical tests were performed using GraphPad Prism version 8.0, IBM SPSS Statistics version 19.0 and Student's t-test.

For manuscripts utilizing custom algorithms or software that are central to the research but not yet described in published literature, software must be made available to editors and reviewers. We strongly encourage code deposition in a community repository (e.g. GitHub). See the Nature Portfolio guidelines for submitting code & software for further information.

Data

Policy information about availability of data

All manuscripts must include a data availability statement. This statement should provide the following information, where applicable:

- Accession codes, unique identifiers, or web links for publicly available datasets
- A description of any restrictions on data availability
- For clinical datasets or third party data, please ensure that the statement adheres to our policy

All the data generated in this study are available with this manuscript and its Supplementary Information. Source data are provided with this paper, and have been deposited in Figshare; DOI: https://doi.org/10.6084/m9.figshare.25097423.

Research involving human participants, their data, or biological material

Policy information about studies with human participants or human data. See also policy information about sex, gender (identity/presentation), and sexual orientation and race, ethnicity and racism.

Reporting on sex and gender	The human participants in breast cancer test had the following sex and gender characteristics: 45 female. The human participants in Epstein-Barr virus test had the following sex and gender characteristics: 63 male and 64 female.
Reporting on race, ethnicity, or other socially relevant groupings	All human participants are Asian.
Population characteristics	The age range of human participants in breast cancer test is 32 to 75. The age range of human participants in Epstein-Barr virus test is 30 to 60.
Recruitment	Inclusion criteria for patients was at least 18 years of age. All serum samples were randomly selected from a larger cohort and analyzed. Unblinding of clinical parameters and corresponding experimental data was performed only after completion of all experiments.
Ethics oversight	All serum samples were collected with the informed consent of the patients, and the study was conducted with the approval of the Internal Review Boards of the designated hospital. Serum samples were obtained from The First Affiliated Hospital, Sun Yat-sen University. No participants were recruited to this study, therefore compensation was not applicable.

Note that full information on the approval of the study protocol must also be provided in the manuscript.

Field-specific reporting

Please select the one below that is the best fit for your research. If you are not sure, read the appropriate sections before making your selection.

☒ Life sciences ☐ Behavioural & social sciences ☐ Ecological, evolutionary & environmental sciences

For a reference copy of the document with all sections, see nature.com/documents/nr-reporting-summary-flat.pdf

Life sciences study design

All studies must disclose on these points even when the disclosure is negative.

Sample size	Inclusion criteria for patients was at least 18 years of age. The sample sizes for breast cancer test (n = 45) and the Epstein-Barr virus test (n = 127) were chosen to achieve statistical significance in the compared endpoints. According to the published studies in the same field, the sample sizes we selected were sufficient to give statistical significance. The results produced by these sample sizes do not affect the analytical decisions of subsequent experiments.
Data exclusions	No data were excluded.
Replication	The experiments were performed in two to three independent replicates as properly indicated in the figure displays or figure legends. The replications had similar and consistent results in our research.
Randomization	Samples were randomly allocated.
Blinding	The experiments were blinded to the operator.

Reporting for specific materials, systems and methods

We require information from authors about some types of materials, experimental systems and methods used in many studies. Here, indicate whether each material, system or method listed is relevant to your study. If you are not sure if a list item applies to your research, read the appropriate section before selecting a response.

Materials & experimental systems

n/a	Involved in the study
☒	☐ Antibodies
☐	☒ Eukaryotic cell lines
☒	☐ Palaeontology and archaeology
☒	☐ Animals and other organisms
☒	☐ Clinical data
☒	☐ Dual use research of concern
☒	☐ Plants

Methods

n/a	Involved in the study
☒	☐ ChIP-seq
☒	☐ Flow cytometry
☒	☐ MRI-based neuroimaging

Eukaryotic cell lines

Policy information about cell lines and Sex and Gender in Research

Cell line source(s)	HEK293T cell line was purchased from Chinese Academy of Sciences Cell Bank.
Authentication	The cell lines were authenticated by the supplier by using mycoplasma analysis, sterility analysis, and visual analysis for growth determination, morphology and viability.
Mycoplasma contamination	All cell lines tested negative for mycoplasma contamination.
Commonly misidentified lines (See ICLAC register)	No commonly misidentified cell lines were used.

Plants

Seed stocks	*Report on the source of all seed stocks or other plant material used. If applicable, state the seed stock centre and catalogue number. If plant specimens were collected from the field, describe the collection location, date and sampling procedures.*
Novel plant genotypes	*Describe the methods by which all novel plant genotypes were produced. This includes those generated by transgenic approaches, gene editing, chemical/radiation-based mutagenesis and hybridization. For transgenic lines, describe the transformation method, the number of independent lines analyzed and the generation upon which experiments were performed. For gene-edited lines, describe the editor used, the endogenous sequence targeted for editing, the targeting guide RNA sequence (if applicable) and how the editor was applied.*
Authentication	*Describe any authentication procedures for each seed stock used or novel genotype generated. Describe any experiments used to assess the effect of a mutation and, where applicable, how potential secondary effects (e.g. second site T-DNA insertions, mosiacism, off-target gene editing) were examined.*
